# Oral Transmission of L-Type Bovine Spongiform Encephalopathy Agent among Cattle

**DOI:** 10.3201/eid2302.161416

**Published:** 2017-02

**Authors:** Hiroyuki Okada, Yoshifumi Iwamaru, Morikazu Imamura, Kohtaro Miyazawa, Yuichi Matsuura, Kentaro Masujin, Yuichi Murayama, Takashi Yokoyama

**Affiliations:** National Agriculture and Food Research Organization, Ibaraki, Japan

**Keywords:** atypical bovine spongiform encephalopathy, cattle, L-type, prion, oral transmission, L-BSE, prions and related diseases, zoonoses

## Abstract

To determine oral transmissibility of the L-type bovine spongiform encephalopathy (BSE) prion, we orally inoculated 16 calves with brain homogenates of the agent. Only 1 animal, given a high dose, showed signs and died at 88 months. These results suggest low risk for oral transmission of the L-BSE agent among cattle.

The epidemic of bovine spongiform encephalopathy (BSE) in cattle is thought to be caused by oral infection through consumption of feed containing the BSE agent (prion). Since 2003, different neuropathologic and molecular phenotypes of BSE have been identified as causing ≈110 cases of atypical BSE worldwide, mainly in aged cattle. Although the etiology and pathogenesis of atypical BSE are not yet fully understood, atypical BSE prions possibly cause sporadic cases of BSE (*1*).

The L-type BSE (L-BSE) prion has been experimentally transmitted to cattle by intracerebral challenge, and the incubation period was is shorter than that for classical BSE (C-BSE) prions (*2**–**6*). The origin of transmissible mink encephalopathy in ranch-raised mink is thought to be caused by ingestion of L-BSE–infected material (*7*). Although L-BSE has been orally transmitted to mouse lemurs (*8*), it remains to be established whether L-BSE can be transmitted to cattle by oral infection. We therefore investigated the transmissibility of L-BSE by the oral route and tissue distribution of disease-associated prion protein (PrP^Sc^) in cattle. All experiments involving animals were performed with the approval of the Animal Ethical Committee and the Animal Care and Use Committee of the National Institute of Animal Health (approval nos. 07–88 and 08–010).

## The Study

We divided a group of 16 Holstein female calves, 3–5 months of age, into 4 groups of 2–6 animals each. Each group of calves was orally administered 1 g (n = 4), 5 g (n = 6), 10 g (n = 4), or 50 g (n = 2) of pooled whole-brain homogenate prepared from cattle experimentally infected with L-BSE (*3**,**6*) (Table). The endpoint titer of the pooled brain homogenate assayed in bovinized transgenic (TgBoPrP) mice was 10^6.9^ of 50% lethal dose/g tissue (data not shown). As noninfected controls, 3 female calves were obtained at 3–4 months of age and euthanized at 60, 92, and 103 months of age, and samples were analyzed as for the experimental animals. 

**Table:** Experimental oral inoculation of 16 calves with brain homogenate of L-type bovine spongiform encephalopathy prions*

Inoculum dose, g	No. inoculated	No. died (postinoculation mo)	No. euthanized (postinoculation mo)
1	4	0	2 (51), 2 (52)
5	6	0	1 (54), 2 (70), 1 (73), 1 (75), 1 (82)
10	4	0	1 (74), 1 (81), 1 (85), 1 (86)
50	2	1 (88)	0

*Necropsies were performed on all animals except the 1 still alive as of December 2016.

At 88 months after inoculation, 1 of the animals (91 months of age) that had received 50 g of L-BSE–infected brain homogenate was unable to get up. The animal extended her forelimbs and hind limbs rigidly forward but did not show persistent knuckling of her fetlock; she did not have difficulty eating and drinking. Seven days after appearance of clinical signs, the animal was found dead, having shown no characteristic signs of L-BSE, such as dullness, lowering of the head, and overreactivity to external stimuli, which had previously been observed after intracerebral inoculation of animals under experimental conditions (*4*).

**Figure 1:**
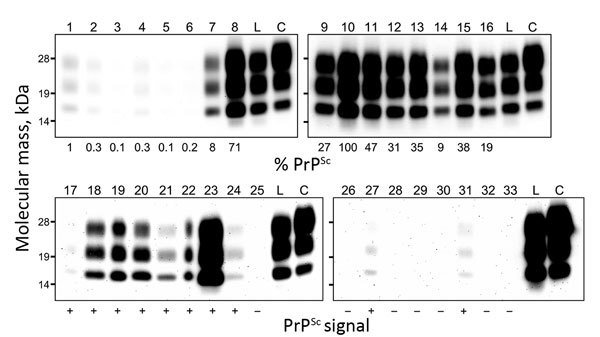
Western blot analysis of proteinase K–resistant disease-associated prion protein (PrP^Sc^) in tissue samples obtained from a cow at 88 months after oral inoculation with brain homogenate of L-type bovine spongiform encephalopathy (BSE) agent. The tissues tested are shown by lane: 1, olfactory bulb; 2, frontal cortex; 3, piriform cortex; 4, parietal cortex; 5, occipital cortex; 6, hippocampus; 7, putamen; 8, thalamus; 9, hypothalamus; 10, midbrain (superior colliculus); 11, obex; 12, cervical enlargement (C7) of spinal cord; 13, lumbar enlargement (L5) of spinal cord; 14, cerebellar cortex; 15, cerebellar white matter; 16, retina; 17, neurohypophysis; 18, trigeminal ganglion; 19, dorsal root ganglion (L5); 20, cervical cranial ganglion; 21, stellate ganglion; 22, celiac and mesenteric ganglion complex; 23, optic nerve; 24, cauda equina; 25, facial nerve; 26, hypoglossal nerve; 27, cervical vagus nerve; 28, sympathetic chain; 29, brachial nerve plexus; 30, sciatic nerve; 31, adrenal gland (medulla); 32, ileum; 33, colon. Lanes 1–16 and lanes 17–33 were loaded with 0.5 mg and 100 mg tissue equivalent, respectively. As controls, lanes L and C were also loaded with 0.5 mg of L-BSE and 0.17 mg of C-BSE cattle brain equivalent, respectively. The relative percentages of PrP^Sc^ (below each lane, upper panel) are normalized against midbrain. The PrP^Sc^ signals in the extracerebral tissues (below each lane, lower panel) are indicated as positive (+) or negative (–).

**Figure 2:**
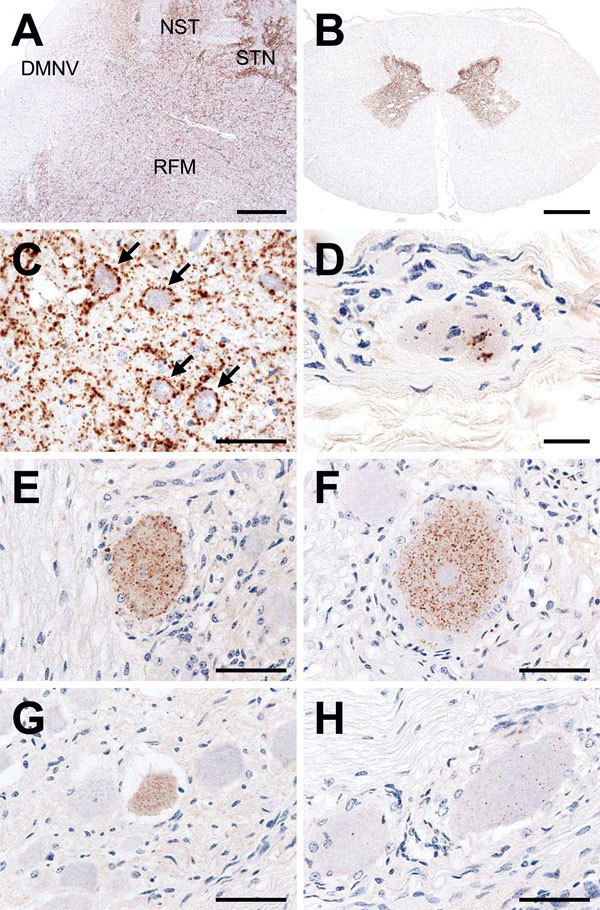
Immunohistochemical detection of disease-associated prion protein (PrP^Sc^) in a cow at 88 months after oral inoculation with brain homogenate of L-type bovine spongiform encephalopathy agent. A) Low amount of PrP^Sc^ deposition in the dorsal motor nucleus of the vagus nerve (DMNV) compared with the more pronounced depositions in the solitary tract nucleus (NST), the spinal tract of trigeminal nerve (STN), and the reticular formation (RFM) in the medulla oblongata at the obex level. Scale bar indicates 200 μm. B) Conspicuous PrP^Sc^ deposition in the gray matter of spinal cord (L6). Scale bar indicates 2 mm. C) Granular staining in the neuropil and perineuronal staining (arrows) in the oculomotor nucleus of the midbrain. Scale bar indicates 50 μm. D) Granular PrP^Sc^ deposition in the muscle spindle of the ocular muscle. Scale bar indicates 20 μm. E–H) PrP^Sc^ in the trigeminal (E), dorsal root (F), cervical cranial (G), and nodose ganglion (H). Scale bars indicate 50 μm.

Histopathologic examination of tissues from this animal revealed minimal or mild spongiform changes of the gray matter neuropil in the thalamic and brainstem nuclei; however, these changes were not visible in the cerebral and cerebellar cortices, the olfactory bulb, or the dorsal motor nucleus of the vagus nerve at the obex. Higher amounts of proteinase K–resistant PrP^Sc^, analyzed by Western blotting with monoclonal antibody T2 (*9*), were detected in the thalamus, brainstem, cerebellum, spinal cord, and retina (Figure 1, lanes 8–16; Figures 2, panels A, B), whereas PrP^Sc^ accumulation was lower in the cerebral cortices and the olfactory bulb (Figure 1, lanes 1–6). The molecular characteristics of proteinase K–resistant PrP^Sc^, such as the molecular weight and the glycoform profile in the brain of the animal, were identical to those observed in the inoculum. The most conspicuous PrP^Sc^ finding, obtained by using immunohistochemistry with monoclonal antibody F99/97.6.1 (VMRD, Pullman, WA, USA), was fine and coarse granular deposits in the neuropil of the thalamus, brainstem, and gray matter of the spinal cord, and in the retina. Perineuronal PrP^Sc^ staining was conspicuous in the large neurons of the thalamic and brainstem nuclei (Figure 2, panel C) but less common in other brain areas. Fewer PrP^Sc^ deposits were dispersed in the dorsal motor nucleus of the vagus nerve at the obex (Figure 2, panel A). No amyloid plaques were detectable in any brain section. In the extracerebral tissues, PrP^Sc^ was lower in most of the samples from the nerve ganglia (trigeminal, dorsal root, stellate, cervical cranial, nodose, and celiac and mesenteric), cauda equina, vagal nerve, optic nerve, neurohypophysis, ocular muscle, and adrenal medulla (Figure 1, lanes 17–33; Figures 2, panels D–H). However, no PrP^Sc^ signal was detected in most of the somatic nerve fibers (Figure 1, lanes 25, 26, 29, 30), the enteric nervous system (Figure 1, lanes 32, 33), and any lymphoid organs including the remaining Peyer’s patches (data not shown).

The only other animal inoculated with 50 g of L-BSE brain material was alive and clinically healthy as of postinoculation month 94 (December 2016). Calves that received 1 g, 5 g, or 10 g of L-BSE brain tissues showed no clinical signs of BSE and were euthanized and underwent necropsy 51–86 months after inoculation (Table). For all of these animals and the uninfected controls, PrP^Sc^ results were negative by Western blot and immunohistochemical analysis. 

## Conclusions

Our results suggest that the risk for oral transmission of L-BSE among cattle may be very low; after 88 months, the only case of transmission occurred in a cow that had been inoculated with a high dose of L-BSE–infected brain homogenate. The incubation period was much longer for cattle dosed orally with L-BSE–infected brain homogenate than for cattle dosed orally with C-BSE–infected tissue (34−74 mo for C-BSE) (*10*). This finding may suggest that the L-BSE prion requires much longer to propagate from the gut to the central nervous system. In addition, the lack of clinical signs, except for difficulty in rising, may present a genuine clinical picture of L-BSE under natural conditions (*11*). In most cases of naturally occurring atypical BSE identified so far, the animals were >8 years of age, except for 3 cases: 1 H-BSE and 1 L-BSE in Spain (*1*) and 1 H-BSE in Germany (*12*). Therefore, we cannot exclude the possibility that L-BSE developed sporadically/spontaneously. However, this case may not have naturally occurred, in view of the low prevalence of L-BSE in Japan during October 2001–August 2016, which was 0.065 cases/1 million tested adult animals. In our study, the remaining live animal, challenged with 50 g of L-BSE brain homogenate, will provide the further information about the oral transmissibility to cattle. Bioassays of brain samples in TgBoPrP mice are ongoing.

The neuroanatomical PrP^Sc^ distribution pattern of orally challenged cattle differed somewhat from that described in cattle naturally and intracerebrally challenged with L-BSE (*2**–**6**,**11**,**13**,**14*), The conspicuous differences between the case we report and cases of natural and experimental infection are 1) higher amounts of PrP^Sc^ in the caudal medulla oblongata and the spinal cord coupled with that in the thalamus and the more rostral brainstem and 2) relatively low amounts of PrP^Sc^ in the cerebral cortices and the olfactory bulb. Furthermore, fewer PrP^Sc^ deposits in the dorsal motor nucleus of the vagus nerve may indicate that the parasympathetic retrogressive neuroinvasion pathway does not contribute to transport of the L-BSE prion from the gut to the brain, which is in contrast to the vagus-associated transport of the agent in C-BSE (*15*). PrP^Sc^ accumulation in the extracerebral tissues may be a result of centrifugal trafficking of the L-BSE prion from the central nervous system along somatic or autonomic nerve fibers rather than centripetal propagation of the agent (*4**,**6**,**9*). Consumption of L-BSE–contaminated feed may pose a risk for oral transmission of the disease agent to cattle.
